# Merkel cell carcinoma and the challenge in its approach: a review based on a clinical context of immunosuppression

**DOI:** 10.1016/j.pbj.0000000000000011

**Published:** 2018-07-03

**Authors:** Filipe Coutinho, Miguel Horta, Estrela Rocha, Carlos Vasconcelos, António Araújo

**Affiliations:** a Service of Medical Oncology; b Service of Dermatology; c Unit of Clinical Immunology; Centro Hospitalar do Porto, Oporto, Portugal

**Keywords:** carcinoma, HIV, immunosuppression, Merkel cell, virus

## Abstract

Merkel cell carcinoma (MCC) is a rare and aggressive neuroendocrine cancer with high rate to local relapse and metastasis. Its connection to immunosuppression is well known, with reported association to human immunodeficiency virus (HIV).

The authors present an 87-year-old woman, infected by HIV type 2 at advanced stage of the disease, whom presented a painless papule on left cheek in 2011. After its total excision, the histopathology confirmed MCC “in situ,” with no regional or distant metastases. Simultaneously, she revealed a viral load of 2220 copies/mL and 224 CD4/mm^3^. Five months later, the patient presented a local and distance relapse with an aggressive behavior and positive regional lymph node. Stage IV disease was confirmed due to presence of liver metastases. Concurrently to the relapse, it was detected low CD4 levels.

In our multidisciplinary team decision meeting, it has been decided conservative treatment due to low Karnofsky status, comorbidities, and stage of disease.

## Introduction

Merkel cell carcinoma (MCC) is a rare and aggressive neuroendocrine skin cancer with a high rate of local recurrence and propensity for regional and distant metastases. It has a high mortality rate, which exceeds the melanoma rates, with a 5-year survival rate ranging from 30% to 64%. It affects predominantly elderly (7th and 8th decades) Caucasian subjects.^1,2^

Several risk factors are being studied as enhancers for this cancer, such as the effect of sunrays exposition and immunosuppressive conditions like post-transplant status, human immunodeficiency virus (HIV) or Chronic Lymphoid Leukaemia.^1^

Few studies correlate the increase of MCC expression with HIV; even though there is a rising evidence that HIV could increase by thirteen times the relative risk of the incidence of this neoplasm.^2^ Nowadays, there are enough data suggesting that Merkel cell polyomavirus (MCPyV) may play an important role in MCC oncogenesis.^3^

With the present case, it is intended to alert to this malignancy in HIV/AIDS patients and to provide clinical tool to its accurate diagnosis and for differential diagnosis with AIDS-related neoplasms.

## Case description

The authors present a case of an 87-year-old Portuguese female, Caucasian, with past risky sexual behavior, and multiple relevant comorbidities, such as Global Initiative on Obstructive Lung Disease stage III chronic obstructive pulmonary disease, cardiac insufficiency, noninsulin diabetes, a monoclonal gammopathy of undetermined significance that never evolved to multiple myeloma, and HIV type 2 infection diagnosed at stage A3 according to Centers for Disease Control and Prevention. She had been followed up in the outpatient ambulatory of the Clinical Immunology Unit since 2004 and had been under antiretroviral therapy (ART) with multiple combinations of drugs such as zidovudine plus lamivudine plus lopinavir/ritonavir, followed by tenofovir plus lamivudine plus atazanavir/ritonavir, and finally due to chronic renal insufficiency, tenofovir was switched to didanosine. The immunological response was not a good one—maximum of CD4 21%, 230/mm^3^—and since 2010 she was never virologically suppressed, translating a bad compliance to ART.

**Figure 1:**
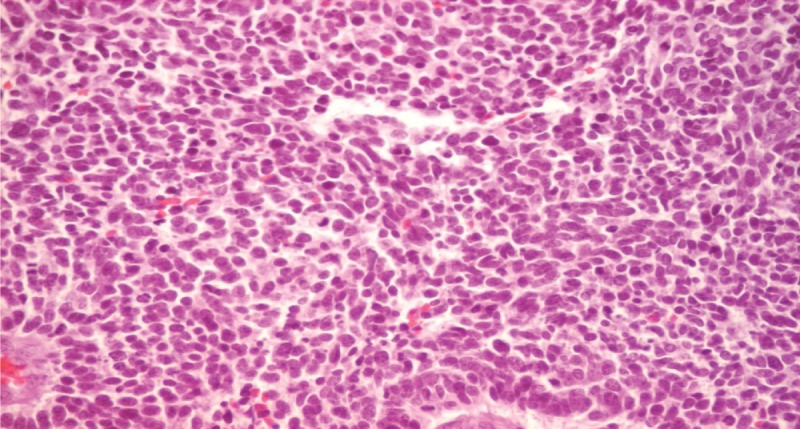
H&E stain, ×40 magnification. The cells with a scant cytoplasm and vesicular nuclei with a salt-and-pepper-like chromatin. Mitotic and apoptotic figures are present.

**Figure 2:**
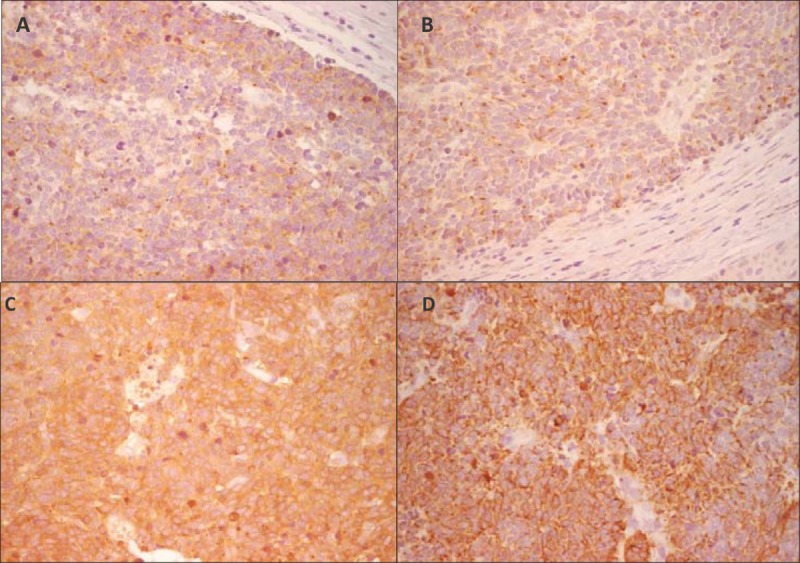
Immunohistochemistry stain, ×40 magnification. It showed positivity to synaptophysin (A), chromogranin A (B), neuron-specific enolase (C), and CAM5.2 (D).

In November 2011, she had been referred by her family physician to a dermatology consultant due to an 8-mm, stiff and painless, erythematous-violaceous papule on left cheek with 2 months of development, without other findings. At first examination, it was difficult to establish a macroscopically identification, which led to consider the differential diagnosis of some AIDS-related neoplasms such as Kaposi Sarcoma or Pseudolymphoma. In an initial approach, it was performed a wide local excision instead of the lesion biopsy. The subsequent histology analysis suggested MCC (Fig. 1) with negative resection margin, and immunohistochemistry (Fig. 2) has confirmed the diagnosis. On clinical examination, it was not obvious lymph node disease and patient did not undergo into sentinel lymph node biopsy. Staging procedures were performed by thoracic, abdominal, and pelvic computed tomography (CT) scan without evidence of regional or distant disease. Thus, the disease was on stage 0. Immunological results detected a viral load of 2200 copies/mL and total CD4 cell of 224/mm^3^, 19%.

**Figure 3:**
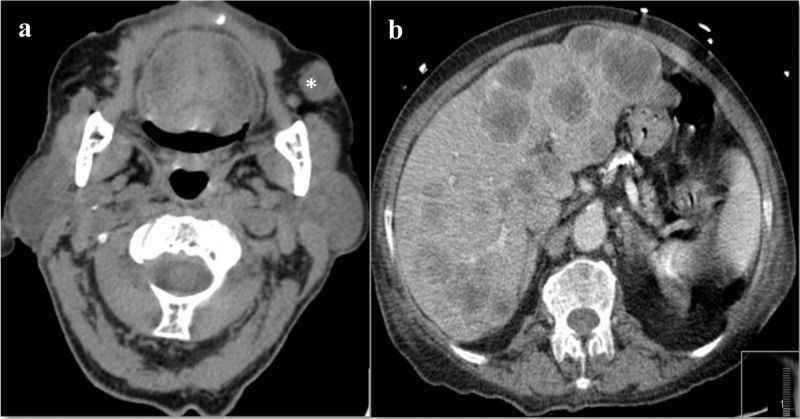
CT scan images at relapse of disease (5 months after diagnosis). Head CT scan (A) showing local tumor aggressiveness with infiltrative pattern (∗). Abdominal CT scan with multiple liver metastases.

In April 2012, the patient has shown recurrence of the lesion at the same location. She has presented local pain and tightness of the adjacent skin, demonstrating a significant local aggressiveness. It was confirmed a histological recurrence of MCC with positive margins, a positive cytology of regional lymph node revealing “salt-and-pepper-like” chromatin. A CT scan of the neck showed an infiltrative lesion (Fig. 3A), and the abdomen CT scan, diffuse liver metastases (Fig. 3B). The blood tests revealed falling of CD4 levels (64 cells/mm^3^). Due to lower Karnofsky performance status (about 50%) and stage IV disease, it was decided in our multidisciplinary team decision meeting that the patient was candidate for best supportive care. The patient died in August 2012 due to complications of her end-stage lung disease.

## Discussion

MCC is rare; however, its incidence is increasing partly due to a better life expectancy and higher survival rates in patients with chronic immunosuppressive diseases.^4^

Nowadays, the incidence of MCPyV is estimated to be present from 43% to 100% of MCC cases,^1^ and its DNA was found fully integrated into the genome of an individual tumor before clonally expansion suggesting a role as an enhancer.^5^ As it was demonstrated high levels of MCPyV in HIV-1 positive patients with MCC, the possibility of a connection between the grade of immunodeficiency, high levels of MCPyV and MCC oncogenesis is becoming more real.^5,6^ Wieland *et al* documented this as they showed significantly higher MCPyV DNA loads in HIV-positive men with poorly controlled HIV infection.^7^

In its clinical screening it is important to remember the acronym “AEIOU” (Asymptomatic, Expanding rapidly, Immunosuppression, Older than 50 years, and UV-exposed location).^4^ However, it is relevant to notice that is common that other lesions can have more than one of these characteristics.

On first instance in clinical observation, MCC can present with an unsuspicious shape and physician should make differential diagnosis with other malignancies such as amelanotic melanoma, cutaneous lymphoma, adnexal tumors, squamous cell carcinoma, pyogenic granuloma, or basal cell carcinoma. Thereafter, it is absolutely necessary to perform histological and immunohistochemistry analysis to confirm the diagnosis.^1,4^ If in a clinical evaluation it is difficult to make a diagnosis of MCC, the pathologic analysis is crucial for its identification in the presence of a positive staining for cytokeratin 20, neurofilaments and neuron-specific enolase, and a negative staining for vimentin, thyroid transcription factor 1, and leukocyte common antigen.^1,8^

The 5-year overall survival rate at presentation ranges from 81% at stage I until 11% at stage IV.^9^

Treatment is individualized depending on clinical staging at presentation, and it can include wide surgical excision, Mohs micrographic surgery, radiotherapy, chemotherapy, or best supportive care. At early stages of disease (0, I, and II), a wide surgical excision should be done. Before definitive excision, a sentinel lymph node evaluation should be performed to exclude microscopic disease, particularly if there is no clinical suspicion of lymph node disease. In the presence of lymph node disease, its radiation or dissection should be done. Radiation therapy can be given in adjuvant setting, especially in some conditions such as tumor >1 cm, positive sentinel lymph node biopsy, underlying chronic immune suppression (eg, HIV disease), evidence of lymphovascular invasion, or positive microscopic margins after excision. In stage I, II, and III, it must be directed into previous lesion site and draining lymph node basin. Radiotherapy can also be given in palliative intent to relieve symptoms such as pain. The benefit of chemotherapy in adjuvant setting was not clearly demonstrated.^8^

Supporting this concept, it was reported a cure of MCC lung metastases after restoration of the immune system with antiretroviral therapy and interleukin 2,^10^ reinforcing the relation between MCC oncogenesis and the immune system. This leads us to assume that the compromise of immunodeficiency control—after the absence of virologic control and immunological response to ART—in our clinical report may have increased the likelihood of progression of disease, regarding the literature.^2^

There is a growing evidence that as much higher the immunosuppressive condition is, more it can negatively influence MCC's survival, as Paulson *et al*^11^ showed in their regression analysis between immune suppressed and nonimmune suppressed patients.

As the immunotherapy is on progressive development in cancer treatment, it is peremptory that these patients should have their immune system restored to provide a viable therapeutic option; nowadays there are ongoing some clinical trials using anti-CTLA-4 drugs such as ipilimumab in nonimmunosuppressed patients.^8^

## Summary

This clinical report illustrates the possibility of the coexistence of MCC in HIV/AIDS patients, probably due to a viral pathway. Thus, it is important to think of it when we are faced with a skin lesion in these patients that AEIOU mnemonic in clinical examination combined by histology analysis is crucial to guide for a definitive diagnosis. The level of immunosuppression may have influence in MCC development and aggressiveness. Thus, the HIV/AIDS treatment efficacy, with good virologic and immunological response, is of most importance to optimize viral rates control and to prevent MCC relapse or progression, providing improved survival. Finally, a multidisciplinary team approach (Dermatology, Medical Oncology, Immunology and Pathology) should be considered to provide an improved clinical assessment to perform an accurate diagnosis, as well to choose the best treatment options.^8^

## Acknowledgments

None.

## Conflicts of interest

The authors declare no conflicts of interest.
